# The Potentials and Caveats of Mesenchymal Stromal Cell-Based Therapies in the Preterm Infant

**DOI:** 10.1155/2018/9652897

**Published:** 2018-04-08

**Authors:** Judith Gronbach, Tayyab Shahzad, Sarah Radajewski, Cho-Ming Chao, Saverio Bellusci, Rory E. Morty, Tobias Reicherzer, Harald Ehrhardt

**Affiliations:** ^1^Department of General Pediatrics and Neonatology, Justus-Liebig-University, Feulgenstrasse 12, 35392 Gießen, Germany; ^2^German Center for Lung Research (DZL), Universities of Giessen and Marburg Lung Center (UGMLC), Feulgenstrasse 12, 35392 Gießen, Germany; ^3^Excellence Cluster Cardio-Pulmonary System (ECCPS), German Center for Lung Research (DZL), Department of Internal Medicine II, Universities of Giessen and Marburg Lung Center (UGMLC), Aulweg 130, 35392 Giessen, Germany; ^4^Department of Lung Development and Remodeling, Max Planck Institute for Heart and Lung Research, German Center Lung Research (DZL), 61231 Bad Nauheim, Germany; ^5^Division of Neonatology, Perinatal Center, University Children's Hospital, Ludwig-Maximilians-University, Campus Grosshadern, Marchioninistr. 15, 81377 Munich, Germany

## Abstract

Preponderance of proinflammatory signals is a characteristic feature of all acute and resulting long-term morbidities of the preterm infant. The proinflammatory actions are best characterized for bronchopulmonary dysplasia (BPD) which is the chronic lung disease of the preterm infant with lifelong restrictions of pulmonary function and severe consequences for psychomotor development and quality of life. Besides BPD, the immature brain, eye, and gut are also exposed to inflammatory injuries provoked by infection, mechanical ventilation, and oxygen toxicity. Despite the tremendous progress in the understanding of disease pathologies, therapeutic interventions with proven efficiency remain restricted to a few drug therapies with restricted therapeutic benefit, partially considerable side effects, and missing option of applicability to the inflamed brain. The therapeutic potential of mesenchymal stromal cells (MSCs)—also known as mesenchymal stem cells—has attracted much attention during the recent years due to their anti-inflammatory activities and their secretion of growth and development-promoting factors. Based on a molecular understanding, this review summarizes the positive actions of exogenous umbilical cord-derived MSCs on the immature lung and brain and the therapeutic potential of reprogramming resident MSCs. The pathomechanistic understanding of MSC actions from the animal model is complemented by the promising results from the first phase I clinical trials testing allogenic MSC transplantation from umbilical cord blood. Despite all the enthusiasm towards this new therapeutic option, the caveats and outstanding issues have to be critically evaluated before a broad introduction of MSC-based therapies.

## 1. Introduction

Inflammatory diseases represent the biggest threat to the preterm infant. They affect all organs including the immature lung, brain, eye, and gastrointestinal tract with extensive and lifelong consequences for the patient's health. So far, efficient therapeutic interventions are restricted to a limited number of drugs and most pathomechanistic insights are available for the inflammatory damage to the immature lung. Therefore, this review is focused on the disease pathology of lung injury and on the therapeutic concepts to protect the immature lung from inflammatory damage. Exogenous mesenchymal stromal cells (MSCs) exert many positive effects on organ development and regeneration [1] and attenuation of all forms of inflammatory processes [2]. Resident MSCs can play an important role in fibrotic diseases including the lung [3]. Therefore, MSC-based therapies have come into the focus of neonatologists. Here, we summarize the current evidence on resident MSCs and the therapeutic potential of exogenous MSCs to reduce the inflammatory damage to the preterm infant.

### 1.1. Epidemiology of Bronchopulmonary Dysplasia

Bronchopulmonary dysplasia is the chronic lung disease of the preterm infant which affects more than 60% with a gestational age < 28 weeks in the US and more than 30% of infants < 30 weeks in Europe [4, 5]. BPD is defined by the clinical criteria of dependency on oxygen or ventilator support at a corrected age of 36 weeks of gestation [6] with grading into “mild,” “moderate,” or “severe” forms [7]. But even infants not fulfilling these criteria display persisting limitations in lung function later in life. Cohort follow-up data substantiate the inability of lung catch-up growth and the persistence of alterations of pulmonary metabolism into adulthood [8]. The long lasting limitations probably lead to recurrent pulmonary sequelae in older age which resemble a COPD-like phenotype in the animal model [9]. Beyond the consequences for exercise capacity and life expectancy, pulmonary sequelae pose an important threat to the overall quality of life in former preterm infants with a close association between limitations in lung function and disorders of somatic growth and psychomotor development [10].

### 1.2. Distortion of Lung Development by Inflammation in the Preterm Infant

The pathogenesis of BPD is caused by the distortion of physiologic lung development in the critical period of the saccular stage. BPD constitutes a multifactorial disease which is caused by the interaction of a plenty of pre-, peri-, and postnatal factors. Being small for gestational age with intrauterine lung growth restrictions caused by placental insufficiency, smoke-induced injury, or diseases emerging during pregnancy, genetic predisposition and the immaturity of the lung with its insufficiency of anti-inflammatory surfactant production represent important prenatal conditions. Pre- and postnatal infections, mechanical ventilation, and oxygen supply are the central triggers of disease pathology with an overwhelming inflammatory reaction in the immature lung which induces or further aggravates lung injury [11, 12].

**Figure 1:**
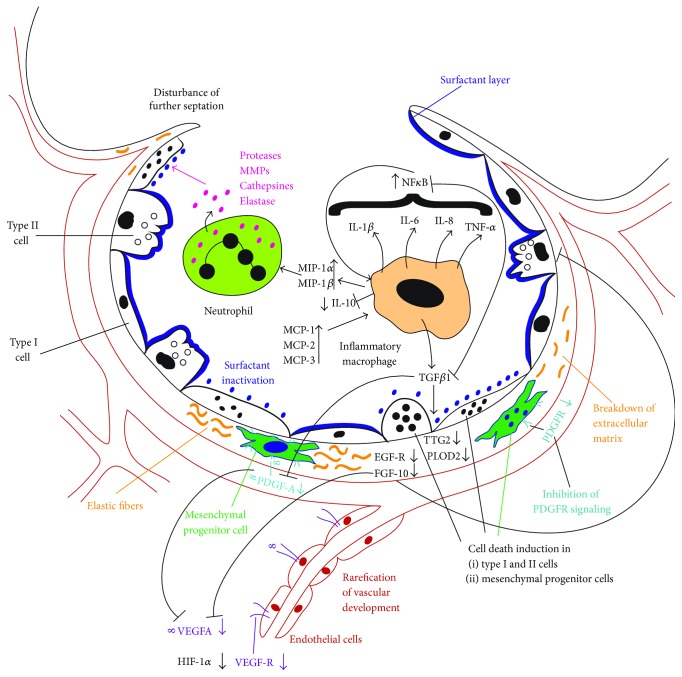
Pathogenesis of BPD: BPD is the consequence of distortion of alveolar, mesenchymal, and vascular development mainly in the saccular stage. Central to the disease is the inflammatory response which is characterized by a disbalance of cytokines and growth factors, the influx of inflammatory cells, cell death induction, and surfactant inactivation. Distortion of mesenchymal progenitor cell function represents a key event in disease pathogenesis.

Characteristic features of lung damage initiation are the overweight of classical proinflammatory cytokines including IL-1*β*, IL-6, IL-8, and TNF-*α* and the absence or downregulation of anti-inflammatory cytokines including IL-4, IL-10, and IL-13 and of lung growth factors including growth factors such as FGFs, VEGFA, and PDGF-A which are required for further physiologic development of the epithelial, mesenchymal, and endothelial compartments of the lung [13]. The resulting leaks in the epithelial barrier promote the influx of inflammatory macrophages and neutrophils which further aggravate lung damage by the release of proinflammatory cytokines and monocyte chemoattractant proteins MCP-1, MCP-2, and MCP-3 and macrophage inflammatory proteins MIP-1*α*, MIP-1*β*, and TGF-*β*1 that augment and sustain the inflammatory reaction [14]. The inflammatory overweight turns the signaling pathway equilibrium into a self-perpetuating disbalance and exaggerated NF*κ*B signaling which boosts inflammation and inflammatory cell survival and aggravates the distortion of the central pathways of lung development. PDGF receptor *α*, bone morphogenetic protein, fibroblast growth factors, PDGFR-*α*, Wnt/*β*-catenin, retinoic acid, VEGFA, and HIF signaling constitute critical pathways affected [15, 16]. Early alterations observed include the inactivation of surfactant proteins and the destruction of organ physiology by cell metaplasia and cell death induction [17]. The characteristic features described are not restricted to the epithelial barrier and alveolar development. Their impact on the mesenchymal and vascular compartment drives the remodeling of the extracellular matrix and the disturbance of vascular development [18]. Further vascular development is disturbed by the inflammatory overweight which suppresses HIF-1*α*, HIF-2*α*, and VEGFA activity [15]. In the mesenchyme, the inflammatory response dysregulates the central pathways of EGF receptor activity, TGF-*β*1-mediated TTG2, PLOD2, and FGF signaling, PDGFR-*α*-dependent VEGFA action, and myofibroblast and mesenchymal progenitor cell function [12, 16, 19–21]. Not surprisingly, dysregulation of myofibroblasts which originate from mesenchymal progenitors and are central drivers of lung development in the saccular stage represents a long-known hallmark of bronchopulmonary dysplasia [22]. Histopathology reveals a diffuse scattering of ACTA-2-positive myofibroblasts in the mesenchyme, and pathologic function is reflected by disturbed growth factor and extracellular matrix component secretion and fibroproliferation [23–26]. Of importance, derangement of any of the three compartments of the epithelium, mesenchyme, and endothelium leads to disruption of lung development [27] and impacts on the others. The reciprocal interplay between the different pathways and the negative consequences of both inhibition or overstimulation of the identical pathway as observed recently for TGF-*β*1 [28, 29], PDGFR-*α*, and NF*κ*B add further levels of complexity but can explain why most promising preclinical therapies failed to prove superiority in the clinical setting [12, 28–33] (Figure 1).

Taken together, the critical hallmark of BPD is the dysregulation of the complex signaling network regulating lung development. Either the activation or inhibition of any central pathway involved in inflammation and/or lung growth results in severe derangement of pulmonary development. Similar pathomechanistic concepts are available for the inflammatory diseases of the cardiovascular system, immature brain, eye, and gut with overlapping and divergent central signaling pathways which have been reviewed in detail [34–39].

### 1.3. Limited Therapeutic Options to Prevent Lung Damage

Despite the progress in clinical care during the last decades, the overall incidence of morbidities of the preterm infant was not reduced due to the steadily increasing survival rates and the successful therapy of more and more immature and small infants [40–42]. Based on the pathomechanistic understanding, far more than 50 different approaches to prevent inflammatory injury or to promote organ development have been tested in clinical trials [43, 44]. In addition, no catch-up growth can be attained later in life [45]. For BPD, special focus has been drawn to the modulation of the pulmonary inflammatory response. Currently, the therapeutic options are restricted to the anti-inflammatory action of corticosteroids and surfactant and the promotion of retinoic acid signaling which are centered during the initial phase of clinical therapy. The efficacy of corticosteroids can be explained by the high potency and the impact on several central pathways of inflammation. Due to the severe side effects on psychomotor development, somatic growth, and endocrinological homeostasis, this therapeutic option is actually restricted to rescue therapy in case of pulmonary failure [46].

Therefore, new therapeutic options based on a molecular understanding are urgently needed. During the recent years, MSCs have gained great interest due to their broad anti-inflammatory and development and repair-promoting properties [47–49]. Therefore, we will first summarize physiologic progenitor cell functions in the preterm infant with special focus on the lung.

## 2. Progenitor Cells in Development and Disease

Progenitor cells play a central role in development and disease. The lung originates from the endoderm and the mesoderm. While the endoderm gives rise to the epithelium, the mesoderm develops into the mesenchyme and endothelium. Lung development is separated into four stages. The pseudoglandular stage with the formation of the first epithelial buds in week 5–16 is followed by the canalicular stage where narrowing of the interstitium takes place. At the border of viability between 22 and 24 weeks of gestation, saccular stage of lung development starts which is a very critical and vulnerable period of lung development. Proper lung development and septation in the saccular stage highly depend on the interaction between the epithelium, the mesenchyme, and the endothelium and the proper localization of mesenchymal progenitors [50]. Currently, we can just speculate on the impact of further cells like alveolar macrophages and the nervous system. Any noxious insult leads to disturbance of this highly orchestrated process. Epithelial and endothelial progenitor cells and MSCs play key roles in this critical period, and any disturbance of their physiologic function leads to disruption of lung development which was repeatedly demonstrated for MSCs and endothelial progenitor cells in vitro and in vivo [51–63].

MSCs are the source for several precursor cells in the immature lung including fibroblasts, lipofibroblasts, and myofibroblasts. They constitute central regulators of lung development in the saccular stage, and their multiple actions during lung development have been reviewed in detail [48, 64, 65]. A special role is ascribed to the production of the main components of the extracellular matrix including elastin and the secretion of the central drivers of lung development including FGF10 [66], Wnt, and PDGF-A [24]. The MSC population is not uniform but constitutes different subsets with cell-specific function in lung development [67–69]. Paracrine secretion by MSCs constitutes a central mechanism of action with impact on alveolar development and angiogenesis. Their antifibrotic and antiapoptotic properties are well accepted for a broad range of diseases [70, 71].

In contrast to terminally differentiated cells, progenitor cells from preterm infants are sensitive to noxious stimuli and more vulnerable than cells from term infants [59, 72] Oxidative stress and hyperoxic exposure of endothelial progenitor cells are associated with reduced numbers circulating in the blood probably due to increased susceptibility towards cell death and accelerated cellular senescence which both can serve as surrogate markers of endothelial dysfunction and distorted vascular development. Furthermore, hyperoxic exposure negatively impacts on paracrine cytokine secretion including VEGFA and NO synthesis [57–61, 63, 72–74]. Epigenetic gene silencing of SIRT-1 was identified to be the critical feature in endothelial progenitors leading to premature p16 stress-induced senescence. Vice versa, biochemical and molecular SIRT-1 rescue experiments provided strong evidence for its central role in prevention of premature senescence of endothelial progenitor cells and the promotion of vessel formation in vitro and in vivo [73]. Comparable insights are described for MSCs and epithelial progenitor cells [75–77]. Taking this into account, the consequences of reduction of progenitor cells following lung injury are well explained. They go along with an overweight of proinflammatory cytokines while protective growth factors and cytokines are deprived [74]. Genetic variants and antenatal factors further predispose to aggravated lung damage [11].

Similar observations are described for the immature brain where neuronal abnormalities and distorted white matter development resulting in hypomyelination are observed together with rarefication of progenitor cell populations. These cells, which are called preoligodendrocytes dominating the preterm brain, are responsible for myelin sheath formation but are extremely sensitive towards inflammatory and hyperoxic injury [78].

Therefore, therapeutic targeting of endogenous progenitor cells and application of exogenous pluripotent cells represent two intriguing strategies to prevent or repair inflammatory damage and to promote organ regrowth.

## 3. Distortion of Resident MSCs in the Pathogenesis of BPD

A decade ago, MSCs were first identified in the tracheal aspirate of preterm infants and were attributed an important role in the development of BPD [79]. MSC-specific characteristic were confirmed by the adherence to uncoated plastic, the expression of mesenchymal cell markers including CD73, CD90, and CD105, the absence of cell surface expression of CD34, CD45, CD11b, CD14, CD19, CD79*α*, and HLA-DR, and the potential of differentiation into adipocytes, osteoblasts, chondrocytes, and myofibroblasts [70, 80]. Overall, studies confirmed the pulmonary origin by cell surface marker expression and RNA profiling and demonstrated phenotype alterations not observed in MSCs derived from other organs and in MSCs from control infants [23]. Recent data proved the presence of MSCs in the tracheal aspirate of every preterm infant [81]. Despite the determination of the pulmonary origin by a lung-specific pattern, the precise origin of these cells from the proximal or distal sections in the lung is not verified [23]. One possible explanation for the appearance of these cells in the alveolar lumen is the promotion of migration by the proinflammatory cytokine overweight induced during lung injury [82]. Three specific changes in the pathway pattern were observed in MSCs from preterm infants later developing BPD. Expression of PDGF receptor *α* was reduced which is a characteristic feature of hypoalveolarization; TGF-*β*1 secretion and *β*-catenin signal transduction were increased which account for myofibroblast differentiation, lung fibrosis, and lung cell death induction. The results from primary MSCs were recapitulated under cell culture conditions when MSCs were exposed to hyperoxia and displayed reduced elastin production and differentiation into myofibroblasts [19, 83, 84]. MSCs obtained from the tracheal aspirates of preterm infants display a proinflammatory phenotype with higher secretion of proinflammatory cytokines including CXCL-1, IL-6, and IL-8 [23]. In a subsequent study, specific changes in MSC phenotype were ascribed a predictive value for the severity of BPD. The MSCs obtained from preterm infants later developing severe BPD displayed more pronounced changes comprising a combined score of cell phenotype, protein expression, and signal transduction. The simultaneous increase in proliferation which was induced by increased NF*κ*B activation was accompanied by the decrease of cellular *α*-SMA levels. The in vitro recapitulation of the inflammatory milieu confirmed the dependency of phenotype alteration on NF*κ*B. The classical proinflammatory cytokines including IL-1*β*, IL-6, IL-8, or TNF-*α* induced the identical changes as observed in MSCs from preterm infants with severe BPD, and NF*κ*B targeting reverted the proinflammatory phenotype [81]. These proof-of-principle data of phenotype reversibility argue for a special focus on pulmonary resident MSC characteristics during the evaluation of any new therapeutic intervention. Vice versa, augmentation of phenotype alteration or increased cell death induction will aggravate lung injury. In a recent animal study, the abrogation of TNF-*α*-mediated NF*κ*B signaling was associated with increased apoptosis induction in PDGFR-*α*-positive mesenchymal cells mediated by predominant TGF*β*1 signaling which explained the aggravated lung damage during mechanical ventilation with oxygen-rich gas [25]. These results are a further example for the complex interplay between the different signaling pathways and the need for a comprehensive evaluation [12, 31].

Taken together, data obtained from human observational studies and in vitro investigations clearly demonstrate that phenotype alterations of resident lung MSCs are associated with the development of BPD. We speculate that reprogramming of resident pulmonary MSCs represents a promising new therapeutic option. Next, we will focus on the therapeutic potential of exogenous MSCs.

## 4. Positive Effects of Exogenous MSCs on the Diseased Lung

During the recent years, a tremendous effort has been undertaken to investigate and optimize stem cell-based therapies in different acute and chronic diseases. Preclinical results mainly from the rodent model [74, 85–91] but also first clinical studies hint to a tremendous potential of stem cell-based therapies to alleviate or cure diseases of the preterm infant [92–94]. MSCs represent the most extensively studied cell population for therapeutic use, and MSCs from different tissues have been tested to prevent the damage to the immature lung or to alleviate the severity of lung damage. Despite the progress in animal studies, the precise molecular mechanisms how these therapies alleviate lung damage remain to be determined. The following chapter summarizes the preclinical evidence available to prevent or treat damage to the preterm lung by exogenous MSC application [71]. The main focus is drawn to give a comprehensive overview of the pathomechanistic understanding based on the studies in the different rodent models. Due to the limitations in space, no critical evaluation of differences in study quality and no separated consideration of MSCs from the umbilical cord blood or bone marrow are presented. We acknowledge the preclinical promising evidence of other cell-based therapies with cell products from the amniotic fluid, Wharton's Jelly, placenta and fetal membranes, and the work with endothelial progenitors and refer to actual comprehensive reviews [95, 96].

MSCs from the umbilical cord or bone marrow have attracted the greatest interest due to the easy accessibility, their multipotency, immune privilege, and low immunogenic potential, and their positive impact on the epithelial, mesenchymal, and vascular compartment of the lung. The first pioneering studies performed in rodents demonstrated beneficial effects of the application of bone marrow-derived exogenous MSCs on survival, lung morphology including alveolar septation, vessel density, and the functional parameters of pulmonary arterial pressure and lung function testing [74, 89, 91, 97, 98]. Beneficial effects were uniformly confirmed in all subsequent studies using the identical approach in the rodent model [85, 87, 88, 99–103]. Identified functions cover all property characteristics for MSC-based therapies and comprise plenty of lung protective mechanisms including lung development promoting anti-inflammatory, antioxidative, and antiapoptotic actions. These results increased survival, the promotion of alveolar and vascular growth, the proper composition of the extracellular matrix, and the inhibition of lung fibrosis [85, 90, 93, 99, 104, 105]. The identical positive effects were demonstrated for the important and frequent clinical situations of perinatal inflammatory exposure or intrauterine growth restriction followed by hyperoxic injury. Both settings revealed identical beneficial effects of MSC transplantation for all areas of interest [106, 107]. The positive effects of exogenous MSCs on lung architecture are not of transient nature, but follow-up examinations confirmed the long-term beneficial effects on inflammatory properties in the lung and lung morphometry. Thereby, the persistent long-term positive effects of both exogenous MSCs despite low engraftment and rapid fade away of the donor cells and of their conditioned media (100, 105) suggest that the paracrine protective effects induced by the transplanted stem cells might initially play a pivotal role in tissue repair (93, 100, 105). As the donor cells fade away, the intact host tissue that is protected by stem cell transplantation might be responsible for the sustained upregulation of various paracrine factors, thus the persistent beneficial effects.

**Figure 2:**
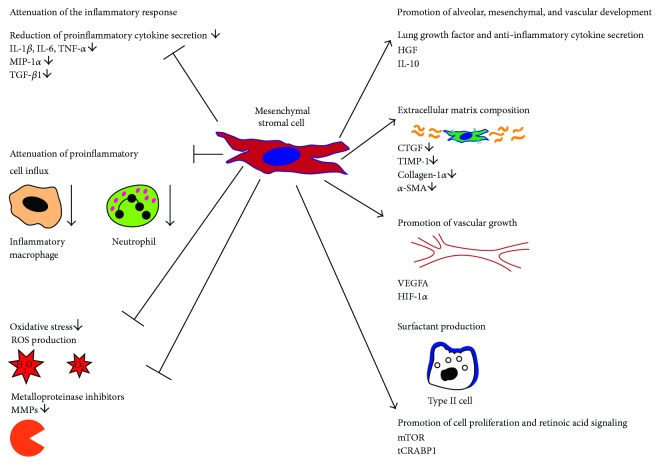
The therapeutic potential of allogenic mesenchymal stromal cells: allogenic MSC transplantation for prevention or therapy of lung injury has been studied in detail in the rodent model. The scheme summarizes all beneficial effects following allogenic MSC therapy with separation of the positive effects on inflammation and organ development.

On a molecular level, MSC therapy leads to a reduction of pulmonary inflammatory response to hyperoxic injury. MSCs have a positive impact on all characteristic proinflammatory features. The classical proinflammatory cytokine levels are reduced including IL-1*β*, IL-6, MIP-1*α*, and TNF-*α*, and the central growth factors and anti-inflammatory cytokines including VEGFA, HGF, and IL-10 are upregulated while TGF-*β*1 levels are decreased. This restoration of cytokine balance leads to an attenuated influx of inflammatory macrophages and neutrophils into the lung. Furthermore, the reduced pulmonary inflammatory response was accompanied by an increase in VEGFA and CTGF signaling and improved lung morphology [106]. MSC transplantation leads to the attenuation of indicators of oxidative stress, reduction of inhibitors of metalloproteinases, adhesion molecules like RANTES, L-selectin, and soluble intercellular adhesion molecule-1 [104], and the preservation of mTOR and tCRABP1 signaling which are key regulators of cell proliferation and retinoic acid pathway, respectively [107]. The last but not the least, application of exogenous MSCs preserves the production of surfactant proteins after hyperoxic injury [91]. The beneficial impact on lung growth with improvements in alveolar and vascular development is accompanied by reduced cell death induction. The reduced expression of genes involved in cell death including the gatekeeper of apoptosis p53 is accompanied by a diminished activation of effector caspases, fewer number of apoptotic cells, and attenuated interstitial lung fibrosis with a decrease in profibrotic genes like CTGF, TIMP-1, collagen-1*α*, *α*-SMA, and TGF-*β*1 [85, 86, 89, 102, 108] (Figure 2).

Besides the direct anti-inflammatory action, data from the bone marrow-derived MSC transplantation argue for a lung self-renewal effect as their application into the lung leads to an increase of bronchoalveolar stem cells. [101]. Experiments in the rodent provide evidence that MSC application contributed to the uprise of decreased growth factors like VEGFA and HGF by paracrine secretion [104, 109]. On a molecular level, RNA interference against VEGFA in exogenous MSCs before application to the injured lung prevented the described increase in VEGFA and concomitantly had a negative impact on all features of hyperoxic lung injury including influx of macrophages, proinflammatory cytokine release, cell death induction, and distortion of alveolar and vascular growth [109] (Figure 2).

The beneficial effects of MSCs are dose dependent, and the transplantation of low numbers of MSCs restricts the positive effects to some of the described MSC features. Different routes of administration have been compared, and intratracheal and intravenous application showed therapeutic efficacy which is in line with the data that stem cells from nonlung tissue can contribute to the attenuation of lung injury [90]. Evidence suggests that the probable dominant mode of action of MSCs is by paracrine effects. The results from animal trials on the other hand suggest also a direct cellular activity which cannot be substituted by cell-free supernatants or exosome preparations and includes mitochondrial transfer. The latter might be of high importance as pathogenesis of BPD is also ascribed to mitochondrial dysfunction [74, 100, 110, 111]. So far, detailed data on the differences between MSC cell-based therapies and application of cell-free extracts, isolated microparticles, or exosomes are missing despite the high enthusiasm about these advanced technologies [105]. Concerns have to be raised with respect to therapeutic equivalence as in the rat hyperoxic model, intratracheal application was superior to the intravenous route. Whether this discrepancy can be ascribed to the dose-response relationship remains unclear. The studies available suggest that the therapeutic benefit of MSC cell therapy is higher for alveolar morphology and lung fibrosis scores which was reproduced by two independent groups [100, 103, 108]. Ex vivo preconditioning might represent a possibility to improve therapeutic efficiency of cell-free extracts [88].

## 5. Further Areas of Research with Exogenous MSC Application

**Figure 3:**
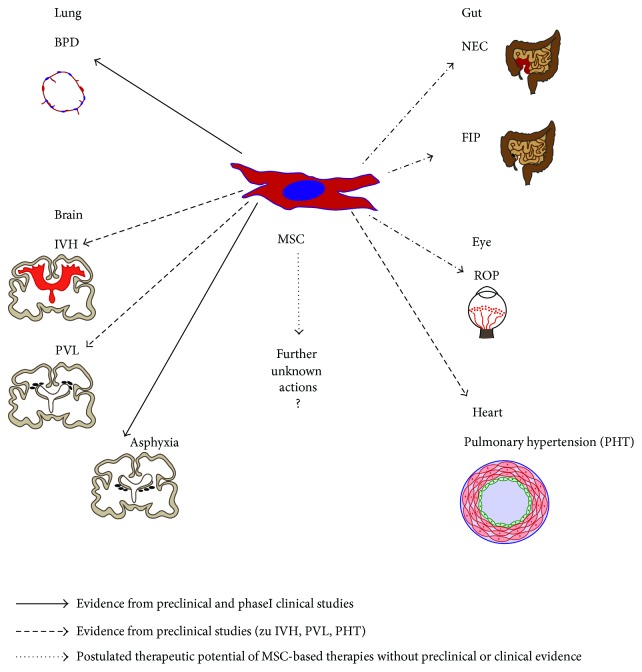
Therapeutic potential of MSC therapy in the preterm and newborn infant: beyond prevention or therapy of lung injury following preterm birth, MSC-based therapies have a therapeutic potential for all inflammatory diseases of the preterm and newborn infant. The scheme summarizes the current status of MSC-based therapies for the lung, brain, gut, eye, and cardiopulmonary system. The scientific knowledge is separated into evidence from phase I clinical trials and preclinical studies mainly in rodents and a presumed therapeutic potential.

Besides the therapeutic potential of MSCs to prevent or reduce the severity of pulmonary sequelae, all other disease entities of the preterm infant with inflammatory properties and loss of cell function are potential targets for cell-based therapies. The current status of MSC-based therapies is summarized in Figure 3.

The central nervous system is the second organ most affected by injury to the preterm infant and therefore has attracted special attention during recent years. Data obtained in preclinical and clinical studies to date suggest a high therapeutic potential of MS-based therapies for cardiovascular diseases, gastrointestinal complications, and retinopathy of prematurity [112–114] which represent further disease entities with dominating inflammatory properties (reviewed in [95]). So far, most preclinical studies in the rodent model were performed for brain pathologies of the term newborn infant including hypoxic brain injury following perinatal asphyxia and neonatal stroke. Actual studies also evaluated the therapeutic potential of MSC therapy in periventricular leukomalacia and intraventricular hemorrhage [115–128]. For intraventricular hemorrhage, the contributions of inflammatory reaction to disease pathologies are well established and include brain damage by cell death induction of neurons and distortion of further myelination and gliosis [34, 129]. In analogy to the inflammatory damage to the immature lung, the selective therapeutic blockade of one inflammatory pathway leads only to a modest or no improvement in brain morphology. In addition, central regulatory pathways of organ damage differ partly from that involved in lung injury as observed for TGF-*β*1 signaling but can be common regulators as for macrophage attraction and proinflammatory cytokine production [129–135]. Therefore, the broader approach of targeting several inflammatory actions as achieved with MSC treatment is reasonable and proven efficient in the rodent model with respect to all relevant brain pathologies including astrogliosis, myelination, cell death induction, and prevention of posthemorrhagic hydrocephalus. Morphologic benefits were accompanied by a strong reduction in classical proinflammatory cytokines like IL-1*α*, IL-1*β*, IL-6, and TNF-*α* and improved performance in behavioral function tests [34, 122]. Brain-derived neurotrophic factor (BDNF) seems to play a pivotal role in the prevention of brain injury, as siRNA knockdown before MSC transplantation abrogated the beneficial effects of MSC transplantation on all morphologic and functional features in the rat model of intraventricular hemorrhage including inflammatory reaction, cell death induction, myelination, astrogliosis, attenuation of posthemorrhagic hydrocephalus, and behavioral test performance [34, 122, 136]. Comparable results were obtained for MSC therapy with cells overexpressing BDNF after hypoxic-ischemic insult and underline the dominant function of BDNF in the brain [137].

The intraventricular route tested in some animal models [120, 122] is restricted by the potential deleterious effects of direct intracerebral injection. Intravenous [138], intracardial [139], and intranasal [119, 125, 127] administration of MSCs proved therapeutic efficiency, and intravenous application revealed similar efficacy with respect to brain morphology parameters and functional read-outs compared to the intraventricular route despite a reduced MSC deposition in the brain tissue [123]. Adjustment of MSC numbers transplanted was a prerogative for comparable efficacy, and scientific data suggest a minimal effective dose for MSC transplantation. In contrast to the lung, repeated application proved superiority with positive effects on the inflammatory response and neuronal apoptosis, brain morphometry, and reactive astrogliosis [118, 119, 128, 140]. Optimal timing is much more critical as the inflammatory reaction following IVH is restricted to a short peak which restricts the therapeutic window [127, 136]. The limited time period can be explained by the cessation of MSC attraction to the site of injury after the short peak of cytokine secretion. In the model of periventricular white matter injury, bone marrow MSCs were attracted from the site of injection to the site of inflammatory injury. The lacking differentiation of these cells into neuronal cells suggests a paracrine neuroprotective effect [120, 124, 127, 141]. As the central nervous system represents a special compartment with reduced immunologic properties, special concerns have to be drawn to the persistent prevalence of MSCs in the brain which has not been studied for the IVH and periventricular white matter injury models so far. In the studies on hypoxic injury, emergence of neoplasia was not detected. [125].

Of importance, the results from the hypoxic ischemic brain injury model suggest that combination of MSC application with the currently practiced evidence-based therapy of therapeutic hypothermia proved superiority compared to each single strategy. The combined approach was the most efficient with respect to the reduction of proinflammatory cytokines, apoptotic cells in the brain, microgliosis, astrocytosis, and functional limitations [121]. These results encourage the pursuit of combinatorial treatment regimens not only in the brain but also in the lung together with therapies with proven efficacy to prevent BPD-like surfactant application.

## 6. MSC Application in Phase I Clinical Trials

A pioneering phase I study to reduce the severity of BPD performed in South Korea was published 3 years ago which demonstrated superiority of exogenous MSC application derived from the umbilical cord blood of healthy newborn infants [94]. MSCs were applied once between days 5 and 14 of life to *n* = 9 infants at high risk for BPD of 24 to 26 weeks of gestation requiring mechanical ventilation and showing deteriorating respiratory function. A significant reduction in BPD severity was observed compared to a control cohort of untreated infants. MSCs were applied in two slightly different dosages which do not allow any conclusion about the optimal number of cells. As expected from the animal experiments, the extent of proinflammatory cytokine response was reduced in the tracheal aspirates following MSC administration and no acute toxicities or side effects were observed until the age of approximately 3 months. The long-term follow-up of safety and efficiency is still pending. Recently, the follow-up at the age of 2 years was published. Despite all the limitations due to the study size, promising data were added for home oxygen, rehospitalization due to pulmonary sequelae, somatic growth, and motor development [92].

In addition to the trials to prevent BPD, two studies on hypoxic ischemic brain encephalopathy and severe intraventricular hemorrhage were conducted during the recent years. The open-labeled study on hypoxic brain injury used autologous umbilical cord blood cells plus therapeutic hypothermia [142]. The evaluation of acute toxicities revealed no acute adverse reactions including cardiovascular compromise or nosocomial infection. The standardized follow-up at the age of 12 months revealed superiority of cell therapy with a combined reduction of neurodevelopmental compromise or death from 59% to 28%. The definite interpretation of the data is hampered by the incomplete follow-up. The enrollment within the IVH study is completed, and no acute toxicities were preliminarily described, but psychomotor follow-up is still awaiting publication [143].

The death of one infant in the MSC group due to sepsis at the age of 4 months in the BPD trial and two additional deaths due to viral infections in the intervention group in the MSC trial for hypoxic ischemic brain injury must be mentioned which occurred in severely handicapped infants [92, 142]. Due to the time period of several months between cell-based therapy and the onset of infection, a direct causal relationship is not probable, but infectious complications should be carefully monitored in the ongoing (follow-up) studies [144].

Taken together, the first phase I MSC trials render promising results and advocate the further evaluation of MSC therapy to reduce the deleterious effects of inflammatory injury to the preterm infant. As these lesions are not restricted to the lung and brain, actual and future studies should carefully consider all other organs prone to inflammatory injury including the cardiovascular system, the gut, and the eye (Figure 3).

## 7. Current Therapeutic Obstacles to MSC Therapy

Despite the tremendous progress in the pathomechanistic understanding of the therapeutic potential of MSC therapy from the rodent models to overcome the deleterious effects of inflammatory diseases in the preterm infant, the broad application of exogenous MSCs to the preterm infant is still hampered by a huge gap of knowledge. Central obstacles include the following: (1) the selection of the most effective cell preparation fulfilling quality criteria which yet have to be defined, (2) the optimal number, time point, and route of MSC cell administration, and (3) the need for validation of clinical criteria to predict the infant at risk benefiting from such a therapy. At present, MSCs represent the most promising candidates for prevention or treatment of BPD and other inflammatory diseases of the preterm infant as MSCs proved therapeutic efficacy in several animal models mimicking BPD, intraventricular hemorrhage, or periventricular white matter injury. In contrast, data on alveolar and endothelial progenitor cells and on epithelial cells from the amniotic fluid [145] display considerable gaps in knowledge with respect to therapeutic efficiency and cell product preparation. Furthermore, experience with the therapeutic potential of endothelial progenitors is restricted to rodent models and might be limited to paracrine effects which can solely rescue vascular outgrowth while epithelial progenitors from the amniotic cavity mainly limit inflammatory processes. MSCs from umbilical cord blood from donor newborn infants can be prepared in sufficient quantity but harbor the risks of allogenic transplantation although the first phase I clinical trials did not show any short-term side effects [94, 142, 146]. Isolation and expansion techniques are still not standardized harboring the risk of variable quality of MSC cell preparations. Currently, MSC differentiation during expansion and related loss of function cannot be monitored by quality control assays, but expansion in cell culture using xeno-free media represents a promising research direction [147]. Furthermore, expansion of autologous MSCs should be taken into the conceptual consideration to overcome the risks and ethical concerns of allogenic transplantation. The application of cell-free supernatants represents an alternative approach to bypass the concerns of allogenic transplantation, but results from animal experiments suggest that the therapeutic efficiency of MSC therapy is mediated by paracrine effects and direct cell interaction at least for BPD [93, 105, 148]. Therefore, MSCs from the umbilical cord blood constitute currently the therapeutic option closest to clinical use. The beneficial effects of comparatively high dosages of MSCs in the rodent model were reproduced in the single phase I clinical trial in infants, but the optimal time point for administration and MSC number remains to be determined. Data from the rodent model suggest that the early preventive application is superior to the late administration to the injured lung and brain. For BPD, actual data do not allow the conclusion that repeated applications are superior to a single dose while repetitive treatments showed superiority for prevention of the deleterious effects of intraventricular hemorrhage [74, 104, 108, 118, 119, 123, 125, 127, 128, 140]. As the majority of preterm infants can be stabilized on noninvasive ventilator support with or without the additional application of surfactant during spontaneous breathing and are generally ventilated much shorter than during the past decades, the intratracheal route of application is restricted to the most severely affected preterms. Uniform distribution of MSCs as ensured for surfactant must be established for any noninvasive application procedure. The intravenous application constitutes an appealing alternative in the preterm infant but has not been studied at all and requires thorough preclinical evaluation. Whether the superiority of intratracheal application of MSCs in the rat model can be attributed to discrepancies in cell numbers delivered to the site of injury remains to be determined. The observed differences in therapeutic efficacy raise concerns about the therapeutic efficiency of the intravenous route [108]. Finally, the inflammatory injuries to the preterm infant result from complex pathophysiologies and resulting disorders currently cannot be predicted based on clinical parameters at birth. While risk calculators can narrow the population at risk, reliable biomarkers are urgently needed for reliable patient selection and monitoring of therapeutic efficiency. The trustworthy selection of infants at high risk is a prerogative for any further therapeutic evaluation of allogenic MSCs in larger scale controlled studies of MSC-based therapies at this stage.

Experimental data on exogenous and resident pulmonary MSCs suggest that both the application of exogenous physiologic cell preparation and the reprogramming of resident pulmonary MSCs represent highly promising therapeutic interventions. While the therapeutic potential of reprogramming resident pulmonary MSCs is still at the very beginning and limited to in vitro investigations, the application of exogenous MSCs requires thorough studies addressing the concerns raised above. One often ignored argument against exogenous MSC preparation is that these cells do not display all properties inherent to resident pulmonary MSCs and that safety issues must be taken into account concerning potential long-term side effects which require decades of research before definitive answers can be given for the preterm situation. It is clearly established that exogenous MSCs do not possess the capability to produce lung growth factors and cytokines and lung-specific proteins including that of the extracellular matrix and do not have the characteristic profile of upregulated transcriptions factors specific for the lung [23, 149, 150]. Special consideration has to be given to the inherent immune system as MSCs can impact on immune cell proliferation and function including dendritic cells, NK cells, and T and B lymphocytes [70, 71, 151]. Therefore, the reports on unexplained deaths due to severe infections as well as the potential risk of malignant transformation in the immune compromised preterm infant have to be evaluated carefully and with respect to the long-term outcome although current data suggest that MSCs in contrast to embryonic stem cells do not display, that is, teratogenic potential even during compromise of the immune system [92, 142, 144, 152]. Finally, it is currently impossible to predict whether genomic instability during MSC expansion is responsible for the altered behavior in vitro during passaging and whether cell propagation gives rise to subpopulations with heterogeneous properties. To overcome these obstacles, MSC-derived supernatants or exosomes represent intriguing alternatives with promising results in first animal trials [153] but as long as it cannot be excluded that cellular MSC components like mitochondria contribute to the protection from inflammatory injury—especially since MSC-derived exosomes showed mitochondria-like aerobic metabolism in one study [154]—a lot of preclinical research activity is needed before safe introduction into clinical trials.

Taken together, MSC-based therapies represent a highly promising research field to ameliorate or overcome the deleterious effects of overwhelming inflammation in the preterm infant. Despite the tremendous progress in pathomechanistic understanding obtained from animal trials during the recent years and the first promising phase I clinical trials, a lot of research effort is needed to bring the optimized MSC product into the upcoming clinical trials. As we have learned from the multicenter studies from the past, the look at the long-term aspects is of central importance for the overall and sustained success of stem cell-based therapies in the preterm infant.
